# Biological Warfare Plan in the 17th Century—the Siege of Candia, 1648–1669

**DOI:** 10.3201/eid2112.130822

**Published:** 2015-12

**Authors:** Eleni Thalassinou, Costas Tsiamis, Effie Poulakou-Rebelakou, Angelos Hatzakis

**Affiliations:** Athens Medical School, University of Athens, Greece

**Keywords:** biological weapons, Candia, Ottoman Empire, plague, Yersinia pestis, Republic of Venice, Crete, bacteria, zoonosis, bioterrorism

## Abstract

A little-known effort to conduct biological warfare occurred during the 17th century. The incident transpired during the Venetian–Ottoman War, when the city of Candia (now Heraklion, Greece) was under siege by the Ottomans (1648–1669). The data we describe, obtained from the Archives of the Venetian State, are related to an operation organized by the Venetian Intelligence Services, which aimed at lifting the siege by infecting the Ottoman soldiers with plague by attacking them with a liquid made from the spleens and buboes of plague victims. Although the plan was perfectly organized, and the deadly mixture was ready to use, the attack was ultimately never carried out. The conception and the detailed cynical planning of the attack on Candia illustrate a dangerous way of thinking about the use of biological weapons and the absence of reservations when potential users, within their religious framework, cast their enemies as undeserving of humanitarian consideration.

## Abstract 2

This incident illustrates how, within a framework of religious fanaticism, the use of biological weapons could appear to be acceptable.

In the course of history, plague, caused by *Yersinia pestis*, has been responsible for at least 3 widespread pandemics with high mortality rates. The first, the “Justinian plague,” spread around the Mediterranean Sea and Western Europe in the 6th century; the second, the so-called Black Death, struck Europe in the 14th century; and the third began in China during the middle of the 19th century and spread throughout the world (*1*–*3*). The Black Death decimated Medieval Europe and had a major effect on the continent’s socioeconomic development, culture, art, religion, and politics (*4*,*5*).

In 1346, plague was deliberately used as a biological weapon. During the siege of Caffa, a Genoese possession in Crimea (now Feodosia, Ukraine), the attacking Mongol forces experienced an epidemic of plague (*6*–*8*). The Mongols, however, converted their misfortune into an opportunity by hurling the cadavers of their deceased into the city, and this action perhaps initiated the ensuing plague epidemic. In 1710, during the Swedish–Russian War, in the siege of Reval (now Tallin, Estonia), the Russians were said to have hurled corpses of plague victims into the besieged city (*9*). During World War II, Japan conducted biological weapons research at facilities in China. Prisoners of war were infected with several pathogens, including *Y. pestis;* >10,000 died as a result of experimental infection or execution after experimentation. At least 11 Chinese cities were attacked with biological agents sprayed from aircraft or introduced into water supplies or food products. *Y. pestis*–infected fleas were released from aircraft over Chinese cities to initiate plague epidemics (*10*). We describe a plan—ultimately abandoned—to use plague as a biological weapon during the Venetian–Ottoman War in the 17th century.

## Archival Sources

Our research has been based on material from the Archives of the Venetian State (*11*). The sources are the communications between the Inquisitors of the State, the Council of Ten, and the commander of Dalmatia. The letters cover the period from February 5, 1649, through August 3, 1651 (i.e., February 5 and 22, 1649; April 1, 21, and 29, 1649; May 9, 1649; December 16, 1650; and August 3, 1651). Although the letters were included in the collections of Venetian documents in works by Lamasky (*12*) and Brown (*13*), they have escaped the attention of medico-historical researchers. 

## Historical Background of Venetian–Ottoman Wars

The siege of Candia, 1648–1669, is the longest in the military history. The city of Candia (now Heraklion, Greece) was the capital of the Kingdom of Candia (Crete) (*Regno di Candia*), which had been a Venetian possession since the fall of Constantinople during the Fourth Crusade in 1204. During the Venetian–Ottoman Wars, the island was the key for the supremacy of the eastern Mediterranean (*14*).

After the fall of Constantinople (1453) and the fall of Rhodes, the possession of the Order of the Knights of Saint John (1522), to the Ottomans, the Republic of Venice was the ultimate protector of Christianity in the eastern Mediterranean. Until the siege of Candia, the 2 superpowers of that era, Venice and the Ottoman Empire, many times crossed swords for the supremacy of the Mediterranean Sea: in 1463–1479, 1499–1502, 1537, and in 1571 in the naval battle of Lepanto (*14*). In addition to the geostrategic and economic reasons, the conflicting religions were another cause of war between Venice and Ottoman Empire. At that time, under the cover of a Holy War, atrocities against the “unbelievers” were a common phenomenon. After so many years of Holy Wars in the area, religious fanaticism was the first instinctive feeling in the mind of the rival armies. 

During the first year of a new war (1644), the Ottoman forces landed in Crete, and the Venetians asked for help from the Pope and other European nations. However, Europe was in the flames of the Thirty Years War (Germany, Austria, and Spain versus Denmark, Sweden, and France). Because England and Holland had commercial agreements with the Ottoman Empire, their leaders refused to help Venice (*15*). In fact, the European efforts to help Candia were disastrous: for example, the expeditions of King Louis XIV of France in 1668 and 1669. The last *provveditore* (governor) of Candia, Francesco Morosini, surrendered the city to the Turks on September 6, 1669, after a blockade of 21 years.

## *Inquisitori di Stato* and the Idea of Biological Warfare

The *Inquisitori di Stato di Venezia* (Inquisitors of the State of Venice) had been established during 1539, after the decision of the *Consiglio dei Dieci* (Council of Ten) to protect the state (*16*). This intelligence service was one of the most effective and deadly in the history of espionage. Venice had an impressive network of spies, and its dark history was connected with political plots, torture, and assassination attempts too numerous to count (*17*).

### Venetian Plan 

On February 5, 1649, the heads of the *Inquisitori*, Piero Morosini, Piero Querini, and Geronimo Giustiniani, received a top-secret letter from Zara (now Zadar, Croatia), a Venetian possession on the Dalmatian Coast. In it, the *Provveditore Generale di Dalmazia et Albania*, Lunardo Foscolo, proposed a plan to end the siege of Candia by infecting the Ottoman forces with a poisonous liquid that he described as “the quintessence of the plague.” The plan was likely to be undetected and successful because plague outbreaks occurred frequently on the island. In fact, since the Black Death pandemic began devastating Europe, 20 outbreaks had occurred on Crete from 1348 through 1645 (*18*,*19*). 

Because an outbreak that occurred only in the Ottoman camp around the city of Candia would be suspicious, Foscolo proposed an alternative “perfect plan”—a massive plague attack against all the Ottoman camps in different places of the island, which would look like a real epidemic of large scale. The plan is detailed as follows (in a typical, Venetian-style letter with long sentences) (*11*–*13*):

To the most Illustrious and most Honored Lords my Masters

My incessant occupation in the discharge of this most laborious service never makes me forget my intent and desire to procure advantage to my country. I then, considering the perilous state of the kingdom of Candia, first treacherously invaded, and now openly occupied by the Turks, the pre-eminence of their forces, the copiousness of their soldiery, the opulence of the Turkish treasury, which will enable them to maintain the war for many years, and also being well aware that, although the public spirit of Venice yields to none in courage and magnanimity, the Republic has neither forces, men, nor money, wherewith to resist much longer the attacks of its foes, and reflecting on the impossibility to meet such a heavy expenditure, have applied myself to a study of the methods whereby the Turkish power might be overcome without risk of men or burden to the exchequer, and how the kingdom of Candia might be recovered; for, after God, our hope to reacquire it is small indeed.

Now there is here a good subject of Venice, lately appointed doctor, who besides his skill in healing is also a famous distiller. His name is Michiel Angelo Salamon. He is desirous to prove himself, what he is in fact, a faithful servant of your Excellencies. I explained my wishes to him, and he availed himself of the presence here of the plague to distil a liquid expressed from the spleen, the buboes, and carbuncles of the plague stricken; and this, when mixed with other ingredients, will have the power wherever it is scattered to slay any number of persons, for it is the quintessence of plague I considered that if this quintessence of plague were sown in the enemies’ camps at Retimo, Cannea, and San Todero, and if it operates as Dr. Michiel assures me it will, this would greatly assist us to recover the kingdom of Candia.

I accordingly determined not to lose the opportunity to have a vase of the poison prepared, and this jar shall be kept, with all due precautions, for the service of your Excellencies. I believe, however, that some ruse must be adopted to entice the Turks into the trap, and would suggest that we should make use of the Albanian fez, or some other cloth goods, which the Turks are accustomed to buy, so that the poison may pass through as many hands in as short a time as possible. The cloth should be made up in parcels as if for sale, after having been painted over with the quintessence, and then placed in separate boxes destined for the various places where we desire to sow the poison. The quintessence, well secured in several cases for the greater safety of those who have to handle and transport it, should be sent to the commander-in-chief that he may take the necessary steps for causing it to pass into the enemies’ hands.

This may be done either by lading several vessels with the cloth, which vessels are to be abandoned by their crews when the enemy comes in sight; or else by means of peddlers who shall hawk the cloth about the country; so that the enemy, hoping to make booty, may gain the plague and find death. The affair must be managed with all circumspection, and the operator must be induced to his work by hopes of gain and by promises, for it will be a dangerous undertaking, and when the operation is over he must go through a rigorous quarantine. While handling the quintessence, it will be of use to the operator to stuff his nose and mouth with sponges soaked in vinegar; and while poisoning the cloth, he may fasten the brush to an iron rod, and when finished, he must put brush and rod into the fire. Having given the Turk the plague, every care must be taken to prevent our people coming in contact with them.

The proposition is a virtuous one, and worthy of the composer of the quintessence. It is, however, a violent course, unusual, and perhaps not admitted by public morality. But desperate cases call for violent remedies, and in the case of the Turks, enemies by faith, treacherous by nature, who have always betrayed your Excellencies, in my humble opinion, the ordinary considerations have no weight.”

In the next letters, the Council of Ten and the *Inquisitori* thank the *provveditore* for his plan and agree that Dr. Salamon, who invented the mixture, should be appointed to carry the poison to the commander-in-chief of the fleet. The commander-in-chief must be warned of the great risk to his own troops from the deadly mixture. However, although Dr. Salamon showed great unwillingness to participate in this operation, the Council of Ten insisted on his presence.

To protect the town of Zara from a possible plague outbreak, the *Inquisitori* further insisted that the cloth goods must be infected on the ship. If, during the voyage, the jar was broken, the crew must empty the contents into the sea. Foscolo succeeded in overcoming Dr. Salamon’s objections, and the appropriate doctor and his jar of quintessence reached the fleet in 1649. Dr. Salamon found Foscolo moving his ships into naval bases to protect them from winter, and he was unable to make use of the mixture at once. Moreover, the commander failed to keep the jar beside him during through the winter. For this reason, Dr. Salamon and “the quintessence of the plague” were once more shipped on board and returned to Zara, likely in late 1649. In Dalmatia, the *Inquisitori,* for safety reasons, put the sample and the doctor in quarantine in a prison. During 1650, Foscolo prepared the Venetian Armada for his attack on Crete and immediately demanded that Dr. Salamon to be sent to him in Candia. The doctor and his jar were liberated, but not before 200 ducats had been exacted from him (as security money for the public property) and given to the Republic of Venice. After Foscolo’s letter about Dr. Salamon’s liberation, on August 3, 1651 (*11*), the story suddenly ended. No further information exists, and probably no further details about this attempt will be known.

## Discussion

Even without knowledge of plague’s microbial nature, the militants of that era understood the “value” of the contamination of an enemy army. The deadly liquid was expressed from the spleens, the buboes, and carbuncles of the victims of a plague outbreak in Dalmatia *(…un liquore scatturito da fieli, bubone et carboni d’appestati con altri ingredient, che averà forza et virtù, dove sarà sparso, essendo la quinta essenze della peste*…). The instructions to the men offer the view of the miasmatic air theory, a theory about the nature of disease that existed as late as the 19th century (*11*,*20*). According to the theories of the era, an infectious disease was the result of the inhalation of miasma (poisonous vapor) of decomposing animal matter, either in the form of aerial emanations or of local pollution of the drinking water by infiltration of such substances. Fundamentally, 2 medical schools of thought existed: those who believed in person-to-person infection and those who believed the existence of a poisonous miasmatic cloud (*21*). Because of the danger of miasma, Foscolo’s men were required to keep their faces covered by sponges soaked in vinegar: “…*l’otturarsi le narici et la bocca con sponga bagnata in aceto…*.”

In terms of beliefs regarding the way the infection could spread, the choice of hats and clothes reflect another issue of that era, that is, the understanding of contaminated objects. Until the 19th century and before the revolution of microbiology, 3 theoretical positions may be distinguished: 1) the miasmatic theory that proposes that contamination is caused by the state of the atmosphere, 2) a modified miasmatic theory that proposes that poor sanitary conditions affect the atmospheric disturbances, and 3) a theory about the combination of miasma/contagion, which may be called contingent contagionism (e.g., that a disease was not contagious in a so-called healthy atmosphere, but might be contagious in an impure atmosphere) (*20*).

After the end of the operation, the authorities had the duty to isolate the peddlers in a pesthouse. This requirement seems logical for the inventors of the quarantine. After the Venetians became a military power, the Venetian Republic soon realized the demographic, economic, and military importance of infectious diseases. The Venetians showed a great interest in preventive medicine and the protection of public health in their possessions (*22*). The Venetian possessions each had their own *proveditore alla sanità* (governor of health), *magistrato alla sanità* (health magistrate), and a *lazaretto* (pesthouse) with its *priore* (director), *dottori* (physicians), and sanitation guards (*23*).

Quarantine of 40 days (from the Italian “quaranta,” meaning 40) was adopted as an obligatory means of isolating persons, animals, and goods that may have been exposed to a contagious disease (*24*). Since the 14th century, quarantine has been the major disease-control strategy, including isolation, sanitary cordons, bills of health issued to ships, fumigation, disinfection, and regulation of groups of persons who were believed to be responsible for spreading the infection (*25*). Also, if the duration of the quarantine is compared with the incubation periods of infectious diseases (e.g., cholera, plague, yellow fever, smallpox), the isolation period overlapped their incubation period (*26*,*27*). During epidemics, the urban health authorities adopted social interventions and traditional health tools, such as quarantine of travelers who had contact with infected persons or who came from a place where the disease was endemic or epidemic (*28*).

The tactics of the so-called dirty war were known to the Venetian army. In Venetian military history, some cases of attacks with chemical agents are recorded (*17*). We suppose that for this reason, Foscolo thought that the Turks would understand the Venetian trick. In the history of the biological war, some cases were complex and results were mixed. A biological attack by the Japanese in 1941 in Changde, China, against the Chinese army and civilians led to 10,000 deaths from cholera in the Chinese population (caused by ingestion of *Vibrio cholera*e–contaminated food and water) but also 1,700 deaths among the unprepared Japanese troops (*29*,*30*).

The Venetian operation would likely have been possible (and not detected) was because of the history of 20 previous plague outbreaks on the island; for this reason, Foscolo proposed the massive attack against the entire island. According to Dr. Salamon and Foscolo, the liquid would have been quite effective; however, this view was not based on the results of an experimental study but on empirical surveillance of the disease’s death rates. 

The main question of the operation, however, was the final efficacy of the mixture. On the basis of current knowledge of *Y. pestis*—the viability of the bacterium outside its normal hosts and its modes of transmission—we do not believe that the Venetian plan would have been effective. How long the bacterium survives outside the host depends greatly on the nature of the material in which it is found. Recent studies evaluating viability of *Y. pestis* on manufactured surfaces (e.g., steel, polyethylene, glass) have shown that survival is typically <72 hours (*31*). Also, the persistence of *Y. pestis* in soil has been suggested as a possible mechanism of interepizootic persistence and epizootic spread and as a factor in defining plague foci (*32*). The studies on plague bacterium survival under natural exposure conditions have shown that *Y. pestis* can survive for at least 24 days in contaminated soil (*33*). In the case described here, the bacteria were unlikely to have remained viable at ambient temperatures for long periods in a distilled solution made from dead host tissues.

The reason the operation was first postponed illustrates another medical theory of that period. Plague was considered a disease of the hot summer “miasmatic” months and was not believed to appear during the winter. 

Unfortunately, we have no information after 1651, but the long interval between the last letters (1650–1651) raises the suspicion that the authorities lost interest in the operation, possibly because of the military and political events during the siege. The Venetian victories in the Aegean Sea (1649, 1651) isolated the Ottoman fleet in Istanbul and left the army in Crete without supplies. Also, the failure provoked angry reactions, and Sultan Mehmed IV changed the leaders of the expedition in Crete. Under those circumstances, the Venetians probably believed that the Ottoman retreat was only a matter of time and postponed the operation.

Concerning the ethics involved in such a plan, Foscolo himself states that this act was violent, unusual, beyond the war rules, and in contrast to the public morality (*…è però violente, insolito et forse non più dalla pieta publica pratticato…*). Even so, the Venetian authorities easily adopted the plan of a massive spreading of the disease. This act could be explained mainly by the religious fanaticism during the cruel Venetian–Ottoman Wars. Also, we must take into consideration the vanity of some leaders who would sacrifice everything to achieve their objectives. As Karl von Klausewitz states in his famous treatise *On War*, “As War is no act of blind passion, but is dominated by the political object, therefore the value of that object determines the measure of the sacrifices by which it is to be purchased” (*34*).

The initial response to deliberate release of infective agents targeted against armies or civilian populations is largely a local responsibility in many parts of the world. To prepare for biological attack, the authorities concerned should be encouraged to make maximum use of existing emergency-response resources. A biological agent attack will generally have the characteristics of an infectious disease outbreak. The respond to a biological incident depends on preparedness (i.e., threat analysis, preparing to response, preparing public information and communication packages, validation of response capabilities) and response (*35*).

Some guidelines have been developed during a crisis and in the absence of experimental data or investigations, such as in the case of the letters filled with a powder containing anthrax (*Bacillus anthracis*) spores in the United States in 2001 (*36*) or the order for smallpox vaccination in Israel during the preparation for the Second Gulf War (*37*). A concept of a modern system of preparedness could be that the risks are not located in the present or in the future but in a shared temporal space and thus can be seen to exist simultaneously (*37*).

In addition, the experience of preparing for bioterrorist attacks can be useful in control of other resurgent infectious diseases and nonbioterrorism emergencies (*38*). For resurgent infectious diseases and microbes classified as agents of biological terrorism, such as *Mycobacterium tuberculosis*, the health care community should have an infection control plan as a part of an overall control program (*39*). In the case of tuberculosis, detecting and curing infective case-patients are the most effective methods of preventing transmission and of controlling the disease in the community (*40*). 

## Conclusions

The use of biological agents as weapons has a long history. In the incident described here, the Venetian authorities in the 17th century adopted a plan for a massive plague attack in Crete to save their possession from Ottoman forces. As we know from the sources, a detailed plan was made for the operation, and the presumably deadly extract from plague victims in Dalmatia was ready for use. The approach of the winter months was an obstacle to operation’s success. According medical thought of that time, plague was a disease of the hot summer months. The Venetians took into consideration the safety of their possessions and, for this reason, they adopted prophylactic measures against plague in their territories. Finally, after 2 years of preparations, they postponed the operation for unknown reasons.

Obviously, according to modern data on the nature of *Y. pestis*, the Venetian plan would not have been effective. In any case, the core of the story is not whether “the quintessence of the plague” would have been effective but the concept of mass destruction through biologic agents. The Venetian plan is another example in the history of biological warfare. In particular, it raises the problem that biological weapons can be used in the name of religious faith, motivated by a deep fanaticism. Unfortunately, throughout history, those driven by this impetus have caused numerous crimes against innocent persons. Such examples prove the need for control and preparedness to ensure national and international safety.
